# Editorial: Novel treatments for diffuse large B-cell lymphoma: The post-CART era

**DOI:** 10.3389/fimmu.2022.1002615

**Published:** 2022-08-08

**Authors:** Alberto Mussetti, Enrico Derenzini, Sarkozy Clementine, Anna Sureda

**Affiliations:** ^1^ Department of Hematology, Catalan Institute of Oncology, Barcelona, Spain; ^2^ Institut d’Investigacio Biomedica de Bellvitge (IDIBELL), Barcelona, Spain; ^3^ Department of Hematology, European Institute of Oncology (IEO), Milan, Italy; ^4^ University of Milan, Milan, Italy; ^5^ Département d’Innovation Thérapeutique et d’Essais Précoces, Gustave Roussy Cancer Campus, Villejuif, France; ^6^ University of Barcelona, Barcelona, Spain

**Keywords:** Lymphoma, CART, cell therapy, bispecific antibodies, monoclonal antibodies

In the last decade, the use of anti-CD19 chimeric antigen receptor T-cells (CARTS) radically changed treatment protocols for large B-cell lymphomas (LBCL) worldwide. In a few years, hematologists learnt how to handle such a therapy and were able to offer a curative treatment to patients who otherwise had no chances of prolonged survival. In the meanwhile, terrific progresses in immunotherapies have also been done. Newer monoclonal antibodies, bispecific antibodies or drug immunoconjugates just follow the discovery of CARTS and open the way to newer therapeutic options for those patients who are not candidates or are refractory or relapsed to anti-CD19 CARTS. There is still a long way to go before declaring diffuse large B-cell lymphoma a completely curable disease. In the post-CART era, immunotherapy has finally gained its *momentum* and the clinical hematologist should be aware of what we learnt so far, where we are and where we are going. In this special issue, we are presenting a series of articles which could serve for this purpose. In the original paper presented by Bastos-Oreiro et al. and the GELTAMO/GETH groups a retrospective comparison of how anti-CD19 CART have higher efficacy than older standard of care therapy against large B cell lymphomas. The population here was composed by refractory/relapsed patients as described by the SCHOLAR-1 study. The authors are clearly showing here the superiority of CARTS compared to therapeutic strategies commonly used in the recent past. Also, an elegant analysis of how the two first anti-CD19 approved CARTS (tisacel and axicel) performed is reported here. Higher efficacy for axicel at cost of higher toxicity is confirmed in this important study which is also showing to the reader the peculiarities of each CART product. In the following paper, Caballero et al. made a superb review of the current state of the art. A summary of the three anti-CD19 CART approved for LBCL is followed by a detailed and comprehensive description of the factors which could impact CART therapy in this setting. A discussion about the patient characteristics (e.g. performance status), tumor features (e.g. preinfusion tumor burden) and CART qualities (e.g. memory-type T cell prevalence, T-cell exhaustion status) is made available to the reader. What happens when the disease is not cured by CAR-T cell therapy? Atallah-Yunes et al. introduced and explained the current clinical scenario of the post-CART setting. A practical and clear view of available immunotherapies is presented here. The authors classified the treatments depending on the surface target antigen, introducing the concept of “surfaceosome” therapy. A list of all the monoclonal antibodies, drug immunoconjugates and bispecific antibodies targeting CD19, CD20, CD22, CD30, CD79b is reported and the reader is left with the ambitious question of how could we combine such therapies in the future. Possibly, the most promising results obtained so far in this setting are related to the use of bispecific antibodies. Barca focused on this newer and promising class of drugs. The structure and the mechanism of action of bispecific antibodies is explained and the results of the pivotal trials of the first anti CD20-CD3 bispecific antibodies mosunetuzumab, glofitamab, epcoritamab and odronextamab are summarized. To conclude, Jeyakumar and Smith closed the topic presenting another highly promising chapter in immunotherapy: allogeneic CARTs. Current CART therapies are technically old, and the arrival of more effective and less toxic cell therapies is expected in the near future. The authors explained the technologies behind allogeneic CARTS, their potential clinical advantages and barriers. We hope that such a view of the post-CART era will be of clinically useful for the Hematology community.

## Author contributions

AM, Manuscript writing and reviewing ED, SC, AS reviewed the manuscript. All authors contributed to the article and approved the submitted version.

## Conflict of interest

The authors declare that the research was conducted in the absence of any commercial or financial relationships that could be construed as a potential conflict of interest.

## Publisher’s note

All claims expressed in this article are solely those of the authors and do not necessarily represent those of their affiliated organizations, or those of the publisher, the editors and the reviewers. Any product that may be evaluated in this article, or claim that may be made by its manufacturer, is not guaranteed or endorsed by the publisher.

